# Impact of baby behaviour on caregiver's infant feeding decisions during the first 6 months of life: A systematic review

**DOI:** 10.1111/mcn.13345

**Published:** 2022-04-01

**Authors:** Mireya Vilar‐Compte, Rafael Pérez‐Escamilla, Dania Orta‐Aleman, Valeria Cruz‐Villalba, Sofía Segura‐Pérez, Kate Nyhan, Linda M. Richter

**Affiliations:** ^1^ Department of Public Health Montclair State University Montclair New Jersey USA; ^2^ Department of Social and Behavioral Sciences Yale School of Public Health New Haven Connecticut USA; ^3^ Human Nutrition Program, Department of International Health Johns Hopkins Bloomberg School of Public Health Baltimore Maryland USA; ^4^ Research Institute for Equitable Development Universidad Iberoamericana Mexico City Mexico; ^5^ Community Nutrition Unit Hispanic Health Council Hartford Connecticut USA; ^6^ Harvey Cushing/John Hay Whitney Medical Library Yale University New Haven Connecticut USA; ^7^ DSI‐NRF Centre of Excellence in Human Development, School of Public Health University of the Witwatersrand Johannesburg South Africa

**Keywords:** breastfeeding, infant crying, infant fussiness, infant sleep, milk insufficiency

## Abstract

Caregivers are often concerned about baby behaviours. Without adequate counselling, parental response can lead to altering infant feeding and jeopardizing breastfeeding. We conducted a systematic review to assess the evidence about the influence of baby behaviours perceived as problematic (crying, sleep waking and posseting) on infant feeding decisions during the first 6 months of life (self‐reported milk insufficiency, breastfeeding duration and introduction of formula). The review focused on quantitative studies published in English, Portuguese or Spanish without date restriction. The search was designed with the support of a medical librarian and conducted in seven databases. Data were managed in Covidence and risk of bias was assessed through the Johanna Briggs Institute critical appraisal checklists. Synthesis of the literature was guided by a conceptual model of the impact of baby behaviours on caregivers feeding practices. We retrieved and reviewed 4312 titles/abstracts and selected 22 for review; 10 were purely descriptive and 12 were cross‐sectional, prospective and quasi‐experimental studies. Although studies from diverse regions were included in the review, more than half were from high‐income countries. All studies reported that baby behaviours affect feeding decisions, the most common baby behaviours studied were crying and fussiness, and the studies suggested relationships with lactation problems and reports of milk insufficiency, maternal breastfeeding confidence, breastfeeding duration and discontinuation, and introduction of formula. There are many factors that lead to perceiving baby behaviours as problematic and there is a need to provide anticipatory guidance to parents and caregivers, starting in pregnancy and counselling through well‐trained health providers.

## Key messages

- Although normal infant behaviour is highly variable, caregivers often perceive infant fussing/crying, posseting and sleep to be problematic.
- How caregivers interpret such ‘problems’ is critical in shaping how they deal with them and the impacts they have on infant feeding practices.
- Developing baby behaviour is not routinely included in training of health care staff, who often misinterpret baby behaviours and advise changes in feeding strategies.
- There is a need for large‐scale studies addressing such issues, as well as clearer guidelines for health professionals and caregivers about common baby behaviours and how to cope with them.

## Graphical abstract

Although normal infant behaviour is highly variable, caregivers often perceive infant fussing/crying, posseting and sleep to be problematic; interpretation of such 'problems' is critical in how they impact on infant feeding practices. Developing baby behaviour is not routinely included in training of health care staff, who often misinterpret baby behaviours and advise changes in feeding strategies. There is a need for large‐scale studies addressing such issues, as well as clearer guidelines.

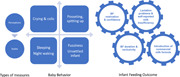

## 1 INTRODUCTION

Up to a third of parents seek help to address concerns about their baby's behaviour in the first few months of life, because they are worried about infant crying, posseting (also referred to as spitting up or spilling) and interrupted sleep (Schmid et al., 2010; Winsper & Wolke, 2014). Unsettled baby behaviour is very distressing to caregivers, as it communicates distress, which causes to instinctively respond with efforts to calm and comfort their infant (Esposito & Bornstein, 2019; Lingle, 2019).

Universally, caregivers contend with unsettled crying, posseting and establishing sleep routines in the first few weeks of life. For example, up to 50% of healthy infants from birth to 3 months of age have at least one episode per day of posseting, peaking at 4 months and then decreasing dramatically to 5% at 10–12 months (Nelson et al., 1997). Sleep–wake regulation evolves rapidly during the first months, but newborns do not have a circadian rhythm and they show disturbances in their sleep until weeks 10–12 when the circadian rhythm begins to emerge (St James‐Roberts & Peachey, 2011). Similarly, the mean fuss/cry duration per day in the first 6 weeks of life established in a review of 28 diary studies, including 8690 infants, was in the region of 2 hours a day. Mean duration dropped rapidly after 6 weeks to about an hour by 10–12 weeks (Wolke et al., 2017).

There is as wide individual variation among infants in these behaviours, as there is among parental responses. Interrupted sleep, posseting and crying often co‐occur (Hudson et al., 2012; St James‐Roberts et al., 1997), partly because crying frequently accompanies both infant waking and regurgitation.

Perceptions of these baby behaviours vary by, among others, cultural values, expectations, self‐efficacy, support and resources (Aktaş & Alemdar, 2019). Parental anxiety frequently determines if baby behaviour is regarded as problematic or not and whether assistance is sought, and few studies find significant correspondence between objective measures and parental report (Douglas & Hill, 2011; Zeevenhooven et al., 2018).

Efforts are made to distinguish between ‘normal’ maturational processes expressed in fussy or unsettled behaviour as infants adapt to conditions outside of the womb, temperamental dysregulation with potential consequences for later parent‐rated child adjustment (Hyde et al., 2012) and clinical conditions requiring treatment. However, even conservative estimates are that fewer than 5% of infants identified by parents to cry excessively are found to have any underlying disease requiring further investigation or treatment, estimated to be <1% of all infants (Freedman et al., 2009). This pattern is similar to sleep problems and posseting, and there is still considerable uncertainty about their causes (Cook et al., 2019).

Among breastfed babies, behavioural cues of fussiness can be interpreted by caregivers, health care staff and family advisors, as indicating that breast milk quality or quantity is inadequate to satisfy the infant (Gussler & Briesemeister, 1980; Mohebati et al., 2021).

The aim of this systematic review is to address the relationship between baby behaviours and feeding practices. More specifically, we are interested in understanding if caregivers are more likely to self‐report insufficient milk, decrease the duration or abandon breastfeeding (BF), or/and introduce commercial milk formulas (CMFs) as a result of problematic crying, infant fussiness, posseting/spitting up and disorganized sleeping/infant waking.

## 2 METHODS

The protocol for this systematic review was registered inPROSPERO (the International Porpsective Register of Systematic Reviews) before starting the literature search (#CRD42021241878). This systematic review followed the guidance of the Preferred Reporting Items for Systematic Reviews and Meta‐Analyses (PRISMA; Moher et al., 2015).

### 2.1 Inclusion and exclusion criteria

Quantitative studies without study design restrictions published in English, Portuguese or Spanish and from low‐, middle‐ or high‐income countries were included if they addressed the following exposures: parental concerns about crying, fussiness, interrupted sleep and posseting/spitting among infants up to 6 months of age. Studies were included only if they focused on healthy mothers and infants who did not have any serious medical conditions or complications from childbirth that could prevent them from breastfeeding or being breastfed. In addition, studies needed to have a comparative perspective on how baby behaviours affect caregiver's infant feeding decisions; this entailed dichotomous, categorical or continuous approaches to assessing the baby behaviour. The outcomes of interest included one or more infant feeding outcomes: exclusive breastfeeding (EBF), any breastfeeding duration or prevalence at different time points, self‐reported insufficient milk (SRIM) and the introduction of CMFs. Table S1 summarizes the inclusion and exclusion criteria.

### 2.2 Search strategy

MEDLINE All, Web of Science Core Collection, PsycINFO and EMBASE, LILACS, Global Index Medicus and Scielo databases were searched electronically for published studies without date restrictions and up to March 2021. The search strategies were designed with support of a medical librarian, using both controlled vocabulary and free text queries, and were tested against a validation set of relevant articles previously selected. The search strategies used terms that fell under the following main concepts: (a) infant feeding and (b) infant crying, or (c) infant fussiness or (d) infant sleep, or (e) infant posseting. To be included in the results of the search, articles needed to have in their title, abstract or text one or more of the following words: breastfeeding, breast milk substitutes, infant formula and prelacteal. The search algorithms are summarized in Table S2.

To try to ensure that no relevant studies were left out, references in included articles were reviewed to determine whether additional articles could be considered. In addition, we reviewed expert researchers’ files to determine additional included articles. This use of both bibliographic databases and citation networks ensured a comprehensive retrieval of relevant articles. The tracking of the search was conducted using Covidence software and the results of the search are presented using the PRISMA flow diagram.

### 2.3 Study selection

To identify the final list of articles for inclusion in the systematic review, the records were uploaded in Covidence. Two of the authors (M. V. C. and D. O. A.) reviewed titles and abstracts of each identified publication and excluded those that did not meet the inclusion criteria. For standardization and consistency, reviewers independently assessed the first 50 titles and abstracts and excluded those that did not meet the inclusion criteria. They then met to review and discuss level of agreement and differences in the operationalization of the inclusion criteria. Following a similar procedure, three authors (M. V. C., D. O. A. and V. C. V.) independently reviewed the full text of articles still considered for inclusion and made a final decision on which ones would be included in the review. For standardization and consistency, reviewers independently assessed the first 10 articles and discussed their decisions. Throughout the process, any differences or questions about the manuscripts’ inclusion were discussed and agreed with two of the other authors (R. P. E. and L. M. R.).

### 2.4 Risk of bias (quality) assessment

The Joanna Briggs Institute (JBI) critical appraisal checklists were used to assess the quality of studies (Munn et al., 2014). The JBI has tools for different study designs, which were accordingly applied for the studies included in the review. Even though the JBI endorses the Grading of Recommendations Assessment, Development and Evaluation approach for systematic reviews and has similar approaches to assessing risk of bias, it has a wider variety of critical appraisal checklists for different research designs, including one of the newest and preferred tools for nonexperimental designs (Ma et al., 2020). It uses a binary scoring process (i.e., yes/no) to assess quality. Three of the authors (M. V. C., D. O. A. and V. C. V.) independently selected the specific JBI critical appraisal checklist corresponding to the study design and assessed the quality of the included articles. Through the binary scores of different domains, the quality assessment of the studies was displayed graphically to highlight their methodological strengths and weaknesses.

### 2.5 Data synthesis

**Figure 1:**
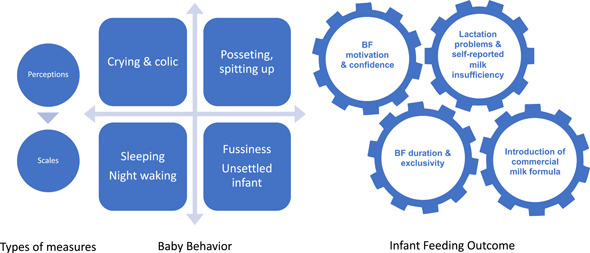
Conceptual model for the impact of baby behaviour on caregiver's infant (>6 m) feeding practices. Original figure developed by the authors

The same authors also extracted the key information from each of the articles using a form that included: authors and year; region, population and setting; sample size; design; dependent, independent and control variables; type of analysis (bivariate/regression, descriptive/comparative); and key findings. For a narrative synthesis of the studies, we followed the conceptual model for the impact of baby behaviour on caregiver's infant (>6 m) feeding practices portrayed in Figure 1.

## 3 RESULTS

### 3.1 Search outcomes

**Figure 2:**
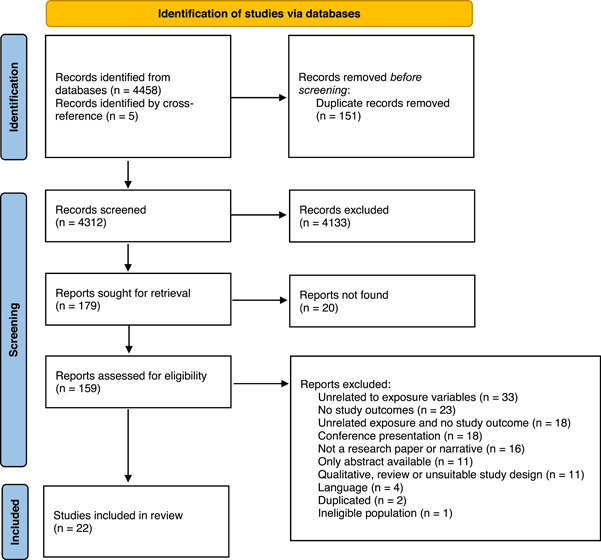
Preferred Reporting Items for Systematic Review and Meta‐Analysis flow chart, baby behaviours and infant feeding systematic review

The search in the 7 databases generated 4458 reports on baby behaviours and infant feeding decisions, with 5 additional records identified by cross‐references (see Figure 2, PRISMA flow chart). After removing 151 duplicates, 4312 titles and abstracts were reviewed. We identified 179 for full‐text review, although 20 documents could not be retrieved. For the remaining 159 articles, a detailed eligibility assessment was conducted, leading to select 22 studies in the current review.

### 3.2 Study characteristics

Of the 22 selected studies, 8 were conducted in North America, 5 in Europe, 3 in the Middle East and North Africa, 2 in the Western Pacific, 2 in Latin America and the Caribbean, 1 in Sub‐Saharan Africa and 1 in East Asia. This implies that 16 were conducted in high‐income, 4 in upper‐middle, 1 in lower‐middle and 1 in a low‐income country (Hamadeh et al., 2021). With respect to study design, 10 studies were purely descriptive, 7 cross‐sectional, 4 prospective and 1 quasi‐experimental. Based on Figure 1, in terms of outcomes, nine studies focused on BF duration and cessation, two on introduction of formula and four addressed early introduction of complementary foods, three on lactation problems and perceptions of milk insufficiency, three on breastfeeding motivation and confidence, and one study addressed both lactation problems and breastfeeding confidence. In terms of the baby behaviours considered, among the 10 descriptive studies, 7 addressed crying and colic‐related behaviours, 4 fussiness and restlessness, and 1 problematic sleeping. These studies are summarized in Table 1. It is important to highlight that these descriptive studies reported only unadjusted prevalence, for which reason we do not assess quality, as there is no formal analysis assessing the association between baby behaviour and infant feeding.

**Table 1:** General characteristics of the 10 descriptive studies related to baby behaviours and infant feeding

No	Author (year), country	Infant feeding outcome	Participants (*n*)	Baby behaviour related to infant decision
1	Kamudoni et al. (2010), Malawi	BF discontinuation	Mothers of infants 0–12 months in urban and rural communities (*n* = 349), analysis referred to EBF discontinuation before 6 months	Most common reason given for EBF discontinuation: ‘crying’ (66.1% semi‐urban, 59.1% rural)
2	Scott & Colin (2002), Australia	BF discontinuation	Women birthing at metropolitan hospitals (*n* = 556)	Most common reason for BF discontinuation: ‘unsettled infant’ (at 2 weeks 37.5%, 3–6 weeks 28.6%, 11–14 weeks 24%, 15–18 weeks 21.7%, 19–24 weeks 14.2%)
3	Bulk‐Bunschoten et al. (2001). The Netherlands	BF discontinuation	Newborns 0–4 months at a well‐baby clinic (*n* = 4438)	Infant‐related reasons for cessation of EBF: ‘crying‐colic’ (19%), perception of a hungry infant (24%)
4	Hernández et al. (1987), Spain	BF discontinuation	Mothers who delivered healthy infants at the General Hospital in Tenerife (*n* = 135)	BF discontinuation due to ‘excessive crying’: 37% at 1 months, 43.4% at 3 months
5	Bloom et al. (1982), Canada	BF discontinuation	BF mothers who delivered healthy babies at Grace Maternity Hospital, Nova Scotia (*n* = 249)	‘Crying and night waking’, assumed to indicate hunger that led to BF discontinuation: 37% at 6 weeks, 29% at 3 months, 14% at 6 months
6	Boban & Zakarija‐Grković (2016), Croatia	Introduction of formula	Mother–infant pairs in a Baby Friendly Hospital Initiative university hospital (*n* = 342)	Most common reason for introducing formula to newborns in‐hospital: ‘crying’ (35.5%)
7	Nevo et al. (2007), Israel	Introduction of formula	Parents of children 6–18 months in a subdistrict of Haifa (*n* = 135), analysis focused on behaviours when babies were 0–6 months	Introduction of formula due to baby's ‘restlessness’ (26%)
8	Segura‐Millán et al. (1994), Mexico	Self‐reported milk insufficiency	Mothers who delivered a healthy baby and were planning to breastfeed in Hermosillo (*n* = 165)	‘Crying baby’ as the reason to self‐report milk insufficiency, 64%–86% of mothers
9	Pastore & Nelson (1997), Canada	BF problems	Mothers at a BF community support centre (*n* = 57)	A ‘crying/fussy baby’ was the reason to visit the clinic for 43% mother with babies < 3 weeks, 47% 3.6 weeks, 40% 7–12 weeks
10	Tracer (2009), Papua New Guinea	BF motivation and confidence	Mother–infant pairs from 6 villages (*n* = 110)	Among ‘fussy babies’, 58% of the mothers reacted to needs, but only 30% through BF

Abbreviations: BE, breastfeeding; EBF, exclusive breastfeeding.

**Figure 3:**
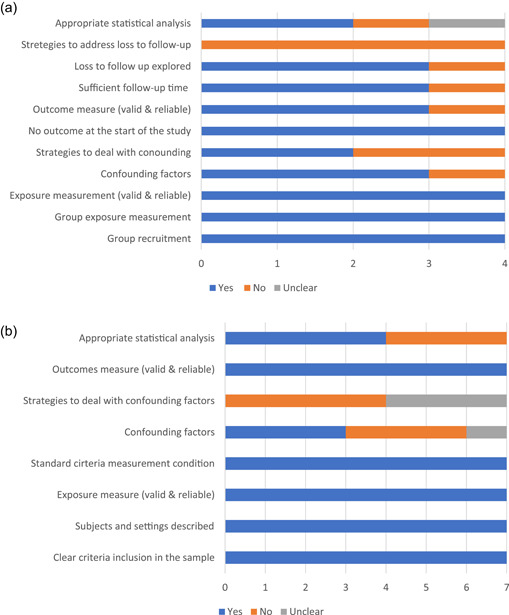
(a) Quality assessment of the prospective cohort studies (*n* = 4) addressing baby behaviours and infant feeding. (b) Quality assessment of the cross‐sectional studies (*n* = 7) addressing baby behaviours and infant feeding

The 12 cross‐sectional, prospective and quasi‐experimental studies are summarized in Table 2. Of these, six studies addressed behaviours related to crying and colic, five to fussiness and restlessness (including infant temperament), and 1 study addressed both. A quality assessment was performed for each study through specific JBI checklists (i.e., analytical cross‐sectional, cohort and quasi‐experimental studies). Figure 3a summarizes the overall quality of the prospective cohort studies and Figure 3b of cross‐sectional ones. The weakest overall quality features for both types of studies is a lack of strategies for dealing with confounding factors, as well as areas of improvement with respect to statistical analyses. In addition, for prospective cohort studies, another weakness was related to strategies to address loss to follow‐up. The only quasi experimental study, had a pre–post design without a control group, which is an important weakness. Although measurement received an overall good assessment, this was mainly driven by clear explanations throughout the studies about operationalization. It is important to highlight that baby behaviours and infant feeding outcomes entail important measurement challenges and mainly rely on perceived reports of caregivers and parent‐rated scales/scores. The majority of the studies (*n* = 8) utilized scales and scores that had previously been evaluated for validity.

**Table 2:** General characteristics of the 12 cross‐sectional, prospective and retrospective studies addressing baby behaviours and infant feeding

No	Author (year), country	Study design: Infant feeding outcome	Participants (*n*)	Baby behaviour related to infant decision
1	Mohebati et al. (2021), Mexico	Prospective. Self‐reported milk insufficiency, BF problems, maternal confidence	Primiparas mothers with healthy and full‐term infants delivered in a Baby Friendly Hospital in Mexico City, planning to breastfeed and who did not undertake paid work until infants were 6 months (*n* = 475)	‘Crying frequency’ associated with ↑ lactation problem score (OR = 1.12), expectation of a baby ‘crying more than other babies’ ↑ reports of milk insufficiency at 2–4 weeks (OR = 2.07). Higher ‘crying expectations’ ↓ maternal self‐confidence (*ρ* = −0.16)
2	Bulut & Alemdar (2021), Turkey	Cross‐sectional. BF motivation	Mothers of infants 3 weeks to 6 months seeking paediatric outpatient care due to excessive crying and who were BF (*n* = 210)	BF enjoyment associated with ↑ positive thoughts (i.e., needs support, trying to communicate) about ‘infant crying’ (*ρ* = 0.235)
3	Wood et al. (2017), USA	One‐group pre–post test pilot intervention. Self‐reported milk insufficiency	Mother–infant dyads followed up postpartum through home visits (*n* = 15)	Counselling home visits associated with ↓ perception of milk insufficiency due to ‘crying’. At 6 days, score attributing milk insufficiency to crying was 2.43 and at 27 days 4.93 (lower score greater attribution)
4	Taut et al. (2016), Ireland	Cross‐sectional. BF duration	Dyads of normal weight, singleton infants and healthy mothers (*n* = 5955)	‘Fussiness/difficultness’ associated with ↓ BF duration (OR = 0.98 at <90 days and OR = 0.98 at ≥90 days)^a^
5	Kronborg et al. (2014), Denmark	Cross‐sectional. Early introduction of complementary foods^b^	Women in urban and rural municipalities who had delivered in the prior 6 months (*n* = 4503)	Only among primipara mothers, perception of infant as ‘not temperamental’ was associated with ↑ odds for introduction of complementary foods after week 25 (OR = 1.77)
6	Keemer (2013), Australia	Cross‐sectional. BF confidence	BF women, singleton, healthy term infant (*n* = 128)	40% of women using second‐line strategies (cup, syringe, bottles, nipples shields) due to ‘unsettled infant’. Using second‐line strategies associated with ↓ BF self‐efficacy (15‐points lower score)
7	Wasser et al. (2011), USA	Cross‐sectional.^c^ Early introduction of complementary foods	Primipara mother–infant dyads enroled in the Supplemental Nutrition Program for Women, Infants and Children (*n* = 217)	Infants perceived to have higher score on ‘temperament scale’ associated with ↑ likelihood to be fed complementary foods at 3 months (OR = 1.97 distress to limitaion, OR = 1.75 activity level)
8	Karaçam (2008), Turkey	Cross‐sectional. Early introduction of complementary foods	Mothers getting services in primary healthcare facilities with babies 0–4 months (*n* = 514)	‘Frequent crying’ associated with ↑ use of complementary foods (OR = 1.687)
9	Howard et al. (2006), USA	Prospective cohort. BF duration	Mother–infant dyads at a university hospital (*n* = 700)	‘Physician‐diagnosed colic’ associated with ↓ duration of breast milk as predominant source of nutrition (hazard ratio = 2.43).
10	Vandiver (1997), USA	Prospective cohort. BF duration	Primiparas married women with healthy newborns (*n* = 50)	Easier ‘infant temperament’ associated with ↑ BF duration (weaned their infants after 12 weeks, *F* = 4.67)
11	Loughlin et al. (1985), USA	Prospective. BF discontinuation	Healthy newborns who were initially breastfed (*n* = 94)	At 8 weeks, 30% of the mothers not BF: rating of nursery staff of infant's ‘excessive crying’ and infant's ‘demanding personality’, associated with BF cessation
12	Forsyth et al. (1985), USA	Cross‐sectional. Early introduction of formula and formula changes	Mothers of BF and FF singleton newborns (BF *n* = 189, FF *n* = 184)	‘Excessive crying and colic’ associated with ↑ introduction of a specialty formula (11% of the BF and 25% of the FF infants given special formulas), mothers believed cause of the problem was intrinsic child (disease, allergy).

Abbreviations: BE, breastfeeding; FF, formula‐fed; OR, odds ratio.

^a^ Data comes from a cohort study that included babies up to 9 months. However, in this particular study, there is a cutoff point showing that fussy babies at 90 days are more likely to have shorter BF duration.

^b^ Although early introduction of complementary foods (i.e., before age 4 months) is not a direct outcome of interest in the review, it is an indirect outcome of BF duration.

^c^ Even though the study was collected prospectively, this study only uses cross‐sectional data from the 3‐month visit.

### 3.3 Study findings

Of the 22 descriptive, cross‐sectional prospective and quasi‐experimental studies, all showed a relationship between problematic baby behaviours and at least one outcome of interest such as reduced maternal breastfeeding confidence, introduction of formula, reduced and/or discontinued breastfeeding. The key findings of the review are summarized through the outcomes indicated in the conceptual model portrayed in Figure 1, illustrating the sequential impacts that baby behaviours can have on infant feeding practices. Although posseting (spitting up or spilling) is mentioned frequently in the literature, we found no studies that met our criteria for inclusion. The main methodological shortcomings of the studies related to sample size, coverage and follow‐up, and a paucity of research on the topic conducted in lower‐middle‐ and low‐income settings.

Common baby behavioural adaptations to the post‐natal environment in the first 6 months of life, including crying, unsettled sleep and posseting, can be distressing to caregivers. The most common interpretation of perceiving that a baby cries too much or wakes up to too frequently at night is that the behaviour results from hunger (Bloom et al., 1982) and/or that infant hunger is related to milk insufficiency (Mohebati et al., 2021; Segura‐Millán et al., 1994; Wood et al., 2017). The literature suggests that crying and fussiness are common problems perceived by caregivers (Pastore & Nelson, 1997; Wood et al., 2017) that, if unattended through adequate counselling, can lead to perceptions of milk insufficiency (Mohebati et al., 2021; Wood et al., 2017), inadequate ways of responding to the infant—such as unwillingness to put the baby to the breast—and reductions in breastfeeding motivation (Tracer, 2009). In addition, there is evidence that among primiparas, having a crying infant is associated with increased lactation problems scores and also a negative correlation with breastfeeding self‐confidence (Mohebati et al., 2021). In contrast, there is evidence that mothers who have a more positive interpretation of crying (i.e., considering it as is a mechanism to communicate and express needs), show a positive correlation with breastfeeding enjoyment (Bulut & Alemdar, 2021). Having an unsettled infant can also affect decisions around the use of second‐line strategies (i.e., syringes, cups, bottles and nipple shields), which have been associated with reductions of up to 15 points on the breastfeeding self‐confidence scale (Keemer, 2013).

It has been found that there is an association between misinterpreting infant adaptive behaviours and decreased maternal confidence and self‐reported milk insufficiency, that in turn increase the risk of early formula feeding or breastfeeding cessation. One study suggested that formula feeding can happen as early as in the birthing facility with a higher prevalence among crying newborns (Boban & Zakarija‐Grković, 2016).

Crying (Karaçam, 2008) and restlessness (Nevo et al., 2007) are common reasons leading caregivers to feed with formula or complementary foods. The complex processes linking baby behaviours, such as crying and fussiness, and infant feeding choices can affect breastfeeding duration and cessation. There is evidence that crying is often the primary reason stated by mothers to stop breastfeeding. This has been reported in low (Kamudoni et al., 2010) and high‐income countries (Bulk‐Bunschoten et al., 2001; Hernández et al., 1987), in urban and rural areas (Hernández et al., 1987; Kamudoni et al., 2010) and at different infant ages (Bloom et al., 1982; Hernández et al., 1987). Crying/colic has been associated with a reduction of breastfeeding as a predominant source of nutrition (Howard et al., 2006).

Nursery ratings and parental perceptions of excessive crying and fussiness (Loughlin et al., 1985) and demanding personality or ‘difficult’ temperament are also associated with breastfeeding cessation (Loughlin et al., 1985), whereas ‘easier’ infant temperaments have been associated with longer breastfeeding duration (Vandiver, 1997). There is, however, no clear evidence if such relationship persists across infants age (Scott & Colin, 2002; Taut et al., 2016). Among breastfed infants, scores profiling more temperamentally ‘difficult’ babies have been associated with increased risk of early introduction to complementary foods (Wasser et al., 2011), whereas perceived ‘easier’ temperaments have been associated with later introduction to complementary foods among primipara mothers (Kronborg et al., 2014). Crying/colic and restlessness can also affect caregiver's beliefs about the health status of the infant, particularly, increasing beliefs and perceptions about allergies and gastrointestinal disorders. This can affect infant feeding choices towards the use of specialty formulas (Forsyth et al., 1985).

## 4 DISCUSSION

Parental perception and interpretations about baby behaviours such as the ones analysed in this review—crying, fussiness, sleeping and posseting—are critical in shaping how caregivers deal with maturing baby behaviours. Our findings are consistent in emphasizing that perceived problems related to these behaviours are distressing to caregivers (Loughlin et al., 1985), who act to ameliorate them, most commonly by changing one or other aspect of infant feeding (Forsyth et al., 1985; Karaçam, 2008). The review reveals that this process is documented in very early stages (Pastore & Nelson, 1997; Scott & Colin, 2002), including during hospital stay after birthing (Boban & Zakarija‐Grković, 2016). It is important to underscore that some normal baby behaviours are now considered to be abnormal, in part because of marketing by the CMFs industry. CMFs frequently use advertisements that appeal to caregivers’ concerns, for example, if a baby is hungry, might have a digestive problem that formula can solve, or can help a baby sleep better (Piwoz & Huffman, 2015). Such claims are not backed by evidence but affect infant feeding choices and undermine breastfeeding confidence (Parry et al., 2013).

However, there is also evidence highlighting that if available, caregivers seek support to address baby behaviours (Pastore & Nelson, 1997), parenting counselling should include pragmatic strategies that caregivers can use to deal with baby behaviours perceived or interpreted as problematic (Wood et al., 2017). A challenge in this respect is that developing baby behaviour is not routinely included in the training of health care staff, who often share parental misinterpretations and advise changing feeds (Karaçam, 2008; Shah et al., 2005). In fact, there are few clear guidelines for health professionals and caregivers about the normal ranges of common baby behaviours and how to cope with them. This is a substantial clinical and public health gap and a responsibility for paediatric and public health researchers to fill it to promote, protect and support optimal feeding for infants.

A limitation of the current review is that we found limited evidence about the association between sleeping and baby behaviour. There is a vast body of literature addressing infant sleeping problems, which emerged in our search, but mainly modelling infant feeding as an explanatory variable of sleep patterns. It is the opposite association that we were seeking to report, namely how problematic sleeping affects infant feeding practices. We also found scarce evidence about the contribution of posseting or spitting up to infant feeding practices. Most of the literature that emerged in the initial steps of the review focused on clinical trials testing different types of formulas. Hence, studies looking at the associations of sleeping and posseting on infant feeding practices should be conducted in the future. In addition, the outcomes used to design the search strategy may have also precluded finding more studies looking at how baby behaviours are associated with the early introduction of complementary foods. It is important to note, however, that although early introduction of complementary foods was not defined as a direct outcome of interest, studies documenting the association of baby behaviours and complementary feeding were detected and included. This was justified for this review, because from a developmental perspective, early introduction of complementary foods is a risk factor for breastfeeding discontinuation (Cohen et al., 1994). Moving forward, it will be important to better understand how early introduction of complementary foods is associated with baby behaviours perceived to be problematic.

In addition, in the review close to half of the studies were purely descriptive (i.e., only providing a description of the baby behaviour and feeding pattern in terms of frequency or prevalence) and there were few high‐quality studies with sufficient sample size and coverage. This suggested that large‐scale studies need to be conducted in low‐, middle‐, and high‐income countries evaluating the contribution that baby behaviours make to infant feeding practices, as well as the relevant role of counselling to support caregivers’ perceptions and strategies to deal with maturational changes in baby behaviour.

A strength of the current review is the inclusion of several baby behaviours and different infant feeding decisions, which were assessed through a substantive conceptual framework. In addition, we included studies in English, Spanish and Portuguese, which is important as systematic reviews that only consider English language publications, which are ubiquitous, are likely to be biased. We are confident that we minimized biasing the review due to language restrictions as we only excluded two abstracts that were only available in French and four papers published in Norwegian, French, Dutch and Japanese, due to language exclusion criteria.

In conclusion, this review provides consistent evidence that baby behaviours such as infant crying and fussiness are distressing to caregivers and are important determinants of infant feeding decision that undermine breastfeeding. Moving forward it is important to provide anticipatory guidance to caregivers since pregnancy, particularly for primipara mothers who might be more uncertain. This will help them to know what to expect regarding baby behaviours and how to manage concerns in partnership with their health providers without undermining breastfeeding. This will also require sound training of health care providers on infant development. It is also important to prevent misinformation presented by the CMFs industry, implying that normal baby behaviours are abnormal and need to be addressed by purchasing their products (Piwoz & Huffman, 2015). This misinformation undermines the breastfeeding self‐efficacy of mothers which in turn increases the risk of SRIM and the introduction of CMF products (Segura‐Pérez et al., 2022).

## CONFLICTS OF INTEREST

The authors declare no conflicts of interest.

## AUTHOR CONTRIBUTIONS

Mireya Vilar‐Compte contributed in conceptualizing and drafting the protocol for the systematic review, reviewed abstracts, titles and manuscripts, and drafted the full manuscript. Dania Orta‐Aleman reviewed abstracts, titles and manuscripts, and participated in synthesis tables. Valeria Cruz‐Villalba reviewed manuscripts and participated in synthesis tables. Sofía Segura‐Pérez contributed in drafting the protocol for the systematic review. Kate Nyhan developed and tested the search strategy, conducted the search, and contributed to defining quality assessment tools. Linda M. Richter and Rafael Pérez‐Escamilla contributed in conceptualizing and drafting the protocol for the systematic review, provided guidance in dissenting and inclusion about specific studies, and supported drafting the manuscript provided a critical review of the full manuscript.

## Supporting information

Suppoting information.

## Data Availability

Since this article is a systematic review, data come from articles in academic journals that have been published in the public domain. Data sharing is not applicable to this article.
