# Supplementary material for: Impact of baby behaviour on caregiver's infant feeding decisions during the first 6 months of life: A systematic review
Source: Matern Child Nutr. 2022 Apr 1;18(Suppl 3):e13345. doi: 10.1111/mcn.13345 (PMC9113474; doi:10.1111/mcn.13345)
Supplement: Supplementary file 1 — Suppoting information. [file MCN-18-e13345-s001.docx]

**Supplementary Materials**

**Table S.1. Inclusion criteria for the impact of baby behavior on caregiver’s infant (>6m) feeding decisions**

| **Criteria** | **Inclusion** | **Exclusion** |
| --- | --- | --- |
| Type of Literature | Peer reviewed journal articles | Non-peer reviewed literature |
| Type of Studies | Quantitative studies without study design restrictions but with a comparison group or exposure | Qualitative studies. Reviews; systematic reviews; meta-analyses (will be used to contextualize review and review list of references) |
| Exposure | Parent concerns about infant crying, fussiness, sleep, and posseting/spitting | Studies not addressing crying, fussiness, interrupted sleep and posseting/spitting as baby behaviors of concern to caregivers |
| Level of Analysis | Caregiver’s infant feeding practices | Studies not reporting neonatal or infant feeding practices |
| Analytical Perspective | Comparative analyses assessing how baby behaviors affect caregiver’s infant (>6m) feeding decisions | Studies without a comparative perspective on how baby behaviors of concern to caregivers affect infant feeding decisions |
| Outcome | Exclusive breastfeeding, any breastfeeding duration or prevalence at different time points, self-reported milk insufficiency or introduction of commercial milk formulas | Studies that do not include infant feeding practice outcomes |
| Target Population | Infants up to 6 months of age who were healthy newborns and whose mothers did not have serious maternal complications from childbirth that would prevent them from breastfeeding | Infants older than 6 months; Newborns with serious medical conditions that prevent them from being breastfed; mothers with complications that prevent them from breastfeeding |

**Table S.2. Search algorithms for the systematic literature search on the selected baby behaviors and infant feeding decisions (infants <6 months of age)**

| 1 | Search |
| --- | --- |
| 2 | [outcome queries: SRIM] |
| 3 | (insufficient adj1 (breastmilk or breast milk or milk)).mp. |
| 4 | ((milk or breastmilk) adj3 dried up).mp. |
| 5 | ((milk or breastmilk) adj3 dry up).mp. |
| 6 | ((baby or babies or infant* or newborn*) adj3 (hungry or “not full”)).mp. |
| 7 | (reason* adj5 (mixed feeding or wean*)).mp. |
| 8 | not enough milk.mp. |
| 9 | not enough breastmilk.mp. |
| 10 | (breastmilk adj5 (early or low or insufficient or sufficient or inadequate or adequate or problems or perceived or perception or volume or supply or production)).mp. |
| 11 | (breastfe* or breast fe* or infant feeding).mp. or exp Infant Nutritionl Physiological Phenomena/ |
| 12 | (milk adj5 (early or low or insufficient of sufficient or inadequate or adequate or problems or perceived or perception or volume or supply or production)).mp. |
| 13 | 3 or 4 or 5 or 6 or 7 or 8 or 9 or 10 or (11 and 12) |
| 14 | Milk, Human/ |
| 15 | exp Lactation Disorders/ or exp Lactation/ |
| 16 | exp infant nutritional physiological phenomena/ |
| 17 | (14 or 15) and 16 |
| 18 | 17 and (early or low or insufficient or sufficient or inadequate or adequate or problems or perceived or perception or volume or supply or production).ti, kf. |
| 19 | 13 or 18 |
| 20 | [outcome queries: infant feeding] |
| 21 | (breast fe* or breastfe*).mp. |
| 22 | (formula or breastmilk substitute* or breast milk substitute*).mp. |
| 23 | BMS.mp. |
| 24 | mixed feeding.mp. |
| 25 | wean*.mp. |
| 26 | infant feeding.mp. |
| 27 | prelacteal*.mp. |
| 28 | exp infant nutritional physiological phenomena/ |
| 29 | milk substitutes/ or infant formula/ |
| 30 | [outcome queries: allergies] |
| 31 | allerg*.mp. |
| 32 | hypersensitiv*.mp. |
| 33 | exp food hypersensitivity/ |
| 34 | allergens/ |
| 35 | or/19-34 [all the outcome queries] |
| 36 | [baby behavior concept] |
| 37 | ((baby or babies or infant* or newborn*) adj3 behavio*).mp. |
| 38 | ((baby or babies or infant* or newborn*) adj5 (crying or cry or cried or fussy or fussiness or fussier or sleep* or posseting or posset or unsettled or distressed or distress or irritability or irritable or consolability or consolable)).mp. |
| 39 | ((hunger or hungry) adj3 (cue* or sign*)).mp. |
| 40 | feeding cue*.mp. |
| 41 | or/37-40 [all the baby behavior queries] |
| 42 | 35 and 41 |
